# Sequential Modulation of Cue Use in the Task Switching Paradigm

**DOI:** 10.3389/fpsyg.2012.00287

**Published:** 2012-08-10

**Authors:** Mike Wendt, Aquiles Luna-Rodriguez, Renate Reisenauer, Thomas Jacobsen, Gesine Dreisbach

**Affiliations:** ^1^Helmut-Schmidt-University/University of the Federal Armed Forces HamburgHamburg, Germany; ^2^University of RegensburgRegensburg, Germany

**Keywords:** task switching, cue validity, sequential modulation, preparation

## Abstract

In task switching studies, pre-cuing of the upcoming task improves performance, indicating preparatory activation of the upcoming task-set, and/or inhibition of the previous task-set. To further investigate cue-based task preparation, the authors presented both valid and invalid task cues in a task switching experiment involving three tasks. Consistent with previous findings, a validity effect in terms of higher reaction times on invalidly compared to validly cued tasks was obtained. However, this validity effect was reduced following invalidly cued trials, suggesting dynamic adjustment in terms of decreased cue-based preparation after being misled. Performance was particularly impaired when the current task was the one that was invalidly cued on the preceding trial. This finding may reflect either particular reluctance to prepare or persisting inhibition of the erroneously prepared task-set from the pre-trial.

## Introduction

In task switching paradigms, participants frequently switch between two or more tasks. Typically the tasks comprise overlapping or identical sets of stimuli, therefore correct task execution critically depends on adoption of the correct task-set. In such situations, performance markedly improves with the option to prepare for the upcoming task. For instance, reaction times (RTs) and error rates decrease when the interval between a cue indicating the identity of the upcoming task and the imperative stimulus increases, more so on task switch than on task repetition (e.g., Meiran, 1996; for a review Kiesel et al., 2010).

Contrasting with the laboratory situation in which pre-knowledge about upcoming task demands can be provided with perfect validity, in real-life settings preparation for an impending activity is almost always associated with some degree of uncertainty. Several studies have addressed the question how the cognitive system deals with such conditions by using advance cues (or task sequence probability) to signal the occurrence of a specific task with differential probabilities. A general finding of such manipulations is that task performance increases with increasing task probability (Ruthruff et al., 2001; Hübner et al., 2004a; Dreisbach and Haider, 2006). Here, we will use informative cues that announce one specific task which – in some (25%) cases – will be followed by a different than the announced task. In the following, we will use the term valid cuing if the cue is followed by the announced task and the term invalid cuing, if the cue is unexpectedly followed by a different task.

The repeatedly observed decreased task performance under conditions of lower likelihood of task occurrence suggests that task preparation is gradually adjusted to its assumed utility. To date, however, still little is known about the mechanism that governs this processing adjustment. One possibility is that the cue usage depends on its recently experienced utility. Such sequential adjustment of cue-based task preparation bears some resemblance with another well documented form of sequential control adjustments. More precisely, reduced response interference from a distractor stimulus feature following trials associated with response conflict has been taken to reflect conflict-induced control adjustment (Botvinick et al., 2001; see also Gratton et al., 1992).

Applying such trial-to-trial adjustment to cue-based task preparation, one might expect that participants engage less in preparation for a cued task after recent invalid cuing. In the current study, we explored this possibility by analyzing task performance as a function of both cue validity on the current and the directly preceding trial.

Invalid task cuing may have additional consequences to possible adjustment of task preparation. One plausible notion is that competition from the erroneously prepared task-set due to invalid cuing may increase and thus trigger extra control measures in terms of reactive inhibition of the “wrong” task-set. In this connection, Hübner et al. (2004a) observed larger task switch costs if the preceding trial involved invalid rather than valid pre-cuing. Based on the assumption of enhanced competition from an erroneously prepared task, the authors attributed this impairment to reactive task-set inhibition (i.e., particular costs of switching to an inhibited task). Because the experiments in that study comprised only two tasks, a task switch following an invalidly cued trial always implied switching to the previously invalidly cued task. Due to this confound it was not possible to decide whether the post-invalid increase of switch cost was indeed due to reactive inhibition of the erroneously prepared task or reflects a general switching impairment after being misled – possibly brought about by reduced preparation. In the current study, we therefore used a task switching paradigm with *three* tasks, which allowed us to compare switching to the task which was erroneously prepared on the previous trial with switching to a different task.

To summarize, the current study investigated aftereffects of erroneous preparation of a task due to invalid cuing. Our main question was whether invalid cuing results in reduced preparatory engagement on the following trial. Furthermore, we wanted to know whether particular costs emerge when switching to a task which was invalidly cued on the preceding trial. In Experiment 1, we administered only task switch trials. In Experiment 2, we extended our investigation to task repetition trials.

## Experiment 1

To investigate current and subsequent consequences of preparation for a not-to-be-executed task, we applied a frequently used task switching paradigm involving three tasks afforded by the same set of stimuli. We presented advance cues which signaled with 75% likelihood the occurrence of a specific task. Because the target stimuli were completely ambiguous regarding the current task, additional information regarding the identity of the relevant task had to be provided in the case of invalid cuing. This was done by presenting a second, coherently valid, task cue, simultaneously with the target stimulus. To ensure that participants did not ignore the advance cues, no simultaneous cues were presented on validly cued trials.

### Method

#### Participants

Fourteen female and six male students of the University of Regensburg participated on a voluntary basis. They ranged in age from 19 to 33 years.

#### Apparatus and stimuli

Participants viewed the screen from a distance of about 60 cm. All target stimuli were presented in white color on a dark gray background and occurred inside a rectangular frame, which was centered on the screen. The digits 1–9, except 5 served as target stimuli. The target stimulus was always presented in the center of the screen and extended 0.7–0.9 cm horizontally and 1.1 cm vertically. Depending on the currently relevant task, participants were instructed to classify the character as odd or even, smaller or larger than five or as extreme or medial (i.e., 1, 2, 8, 9 vs. 3, 4, 6, 7). Responses were given on a standard QWERTZ keyboard. Participants were instructed to press the left key (“y”-key) for *smaller*, and the right key (“m”-key) for *larger*. The S-R assignment in the *odd/even* and the *medial/extreme* tasks was counterbalanced across participants.

### Procedure

On each trial, the task and the target stimulus was chosen randomly with the only constraint that no task was repeated on a subsequent trial. The target stimulus remained on the screen until a response key was pressed. Throughout each block of trials the rectangular frame was shown. It was filled with one of three colors to indicate the upcoming task. On a random 25% of trials this color cue did not match the upcoming task but the other task that was not presented on the preceding trial. Yellow indicated the odd/even task, cyan indicated the smaller/larger task, and purple indicated the extreme/medial task. These task cues were shown 500 ms after a response key was pressed, and remained on the screen for 100 ms, followed by a blank screen (except for the rectangular frame) for 400 ms, after which the target stimulus was presented. In case of an invalid trial, the target stimulus was presented with a simultaneous task cue, “overruling” the advance cue. In case of a validly cued task, no additional task cue was presented with the target stimulus.

Participants were instructed to identify the target by pressing the assigned response key as quickly as possible while avoiding errors. In case of an incorrect response, error feedback occurred for 800 ms slightly below the center of the screen. After three practice blocks of 20 trials each (the first block comprising only odd/even decisions, the second block comprising only smaller/larger decisions, and the third block comprising only extreme/medial decisions), participants were administered 10 blocks of 99 trials each. They were allowed to rest between blocks.

### Results

The first three trials of each experimental block were considered “warm-up” trials and did not enter the statistical analyses. In addition, data from trials with RTs deviating more than 2 standard deviations from the mean RT of each experimental condition per participant were considered outliers and were also excluded from the analyses.

**Figure 1:**
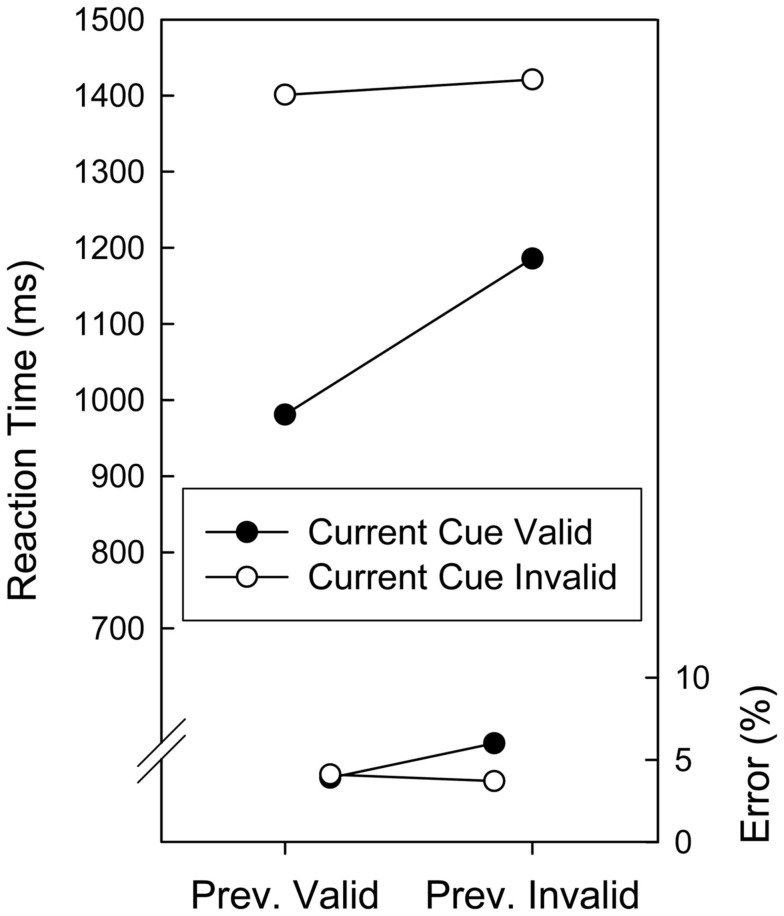
**Mean reaction times and error proportions of Experiment 1 as a function of cuing validity of the current and preceding trials**.

In an Analysis of Variance (ANOVA) with repeated measures on the factors Validity on Current Trial (valid, invalid), and Validity on Preceding Trial (valid, invalid) on the mean RTs, both main effects reached significance. There was an invalidity cost of 328 ms, *F*(1, 19) = 188.35, *p* < 0.001, and post-invalid slowing of 112 ms, *F*(1, 19) = 87.52, *p* < 0.001. As can also be seen in Figure 1, post-invalid slowing was confined to validly cued trials, resulting in a two-way interaction, *F*(1, 19) = 28.21, *p* < 0.001. The invalidity cost amounted to 422 and 235 ms after validly and invalidly cued trials, respectively. The corresponding ANOVA on error rates revealed a significant effect of Validity on the Preceding Trial, *F*(1, 19) = 5.03, *p* = 0.037, and marginally significant effect of Validity on the Current Trial, *F*(1, 19) = 4.28, *p* = 0.053. Both factors entered into an interaction, *F*(1, 19) = 5.51, *p* = 0.030, reflecting that following an invalid trial, invalid trials were associated with less errors than validly cued trials (3.7 vs. 6.0%) whereas there was no difference after validly cued trials (4.1 vs. 3.9%).

**Figure 2:**
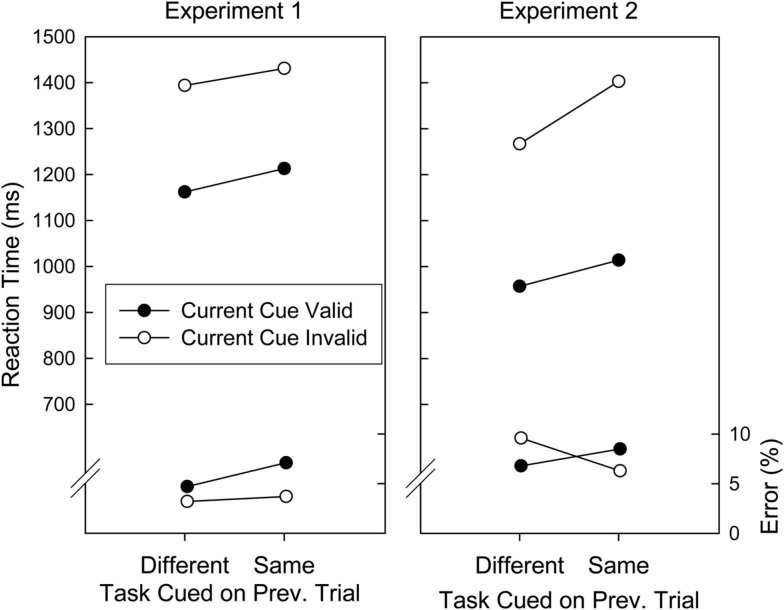
**Mean reaction times and error proportions of Experiment 1 and 2 for task switch trials following an invalidly cued trial as a function of cuing validity and whether the switch was made to the previously (invalidly) cued task**.

To further explore the effects of previous invalid cuing, we ran a second analysis, confined to data from task switch trials following invalidly cued trials, only. More precisely, to find out, whether the observed post-invalid adjustments were further modulated by the specific task that was invalidly cued on the pre-trial, we ran an ANOVA with the factors Validity on Current Trial (valid, invalid) and Task (Invalidly) Cued on Preceding Trial (different, same as current task). The mean RT and error data are depicted in Figure 2, left panel. As can be seen, switching to the task that was invalidly cued on the preceding trial impaired performance in both RTs and errors [*F*(1, 19) = 4.04, *p* = 0.059, and *F*(1, 19) = 5.07, *p* = 0.036, respectively]. This impairment did not differ for validly and invalidly cued trials [*F*(1, 19) < 1, and *F*(1, 19) = 1.27, *p* = 0.273, for RTs and errors].

### Discussion

Experiment 1 replicated costs of invalid task cuing previously found with other procedures (Ruthruff et al., 2001; Dreisbach et al., 2002; Hübner et al., 2004a). Interestingly, in the current study, the invalidity cost was confined to RTs, suggesting that participants adopted a response strategy which ensured constant performance accuracy across valid and invalid cuing conditions. As expected on the assumption of trial-to-trial adjustment of task preparation on the basis of its previous utility, the invalidity cost was reduced when the preceding trial was invalid, suggesting a general reluctance to engage in cue-based task preparation after having been misled.

Performance on trials associated with invalid cuing was particularly impaired when it implied switching to the task which was (invalidly) cued on the preceding trial. This extra cost is consistent with the assumption that an invalidly prepared task-set becomes subject to reactive inhibition. However, an alternative interpretation must be considered which relates to a confound with the specific cue sequence. More precisely, validly cued trials associated with switching to the previously cued task (e.g., aBaA with uppercase letters denoting tasks and lowercase letters denoting cues, respectively), and invalidly cued trials associated with switching to the remaining task (e.g., cBcA) are both necessarily associated with a cue repetition. That is, a lack of preparation following invalid switches should impair performance for valid task switches (aBaA), because here preparation would activate the correct task. Conversely, on invalid switches (following invalid switches, cBcA), in which preparation would activate an incorrect task-set, performance would be improved. Our results are thus also consistent with the assumption that participants are particularly reluctant to engage in cue-based preparation for the task which was invalidly cued on the preceding trial. It might also be conjectured that the lack of preparation is bound to usage of the previously misleading cue. However, the fact that Hübner et al. (2004a, Experiment 4) found increased switch costs after invalid cuing even when different cues were used to indicate the same task, argues against this possibility. Although our data do not allow deciding between reactive inhibition and task-specific lack of preparation, the fact that the invalidity cost manifested only in RTs whereas the extra cost of switching to the previously cued task occurred in both RTs and errors suggests that different processes may underlie the two effects.

## Experiment 2

Experiment 2 closely resembled Experiment 1, the main modification being that, on each trial, the task was chosen randomly, resulting in an expected proportion of one third task repetition trials. The purpose of Experiment 2 was twofold. First, we wanted to replicate the sequential modulation of cuing validity obtained in Experiment 1. Second, extending the procedure to task repetitions allowed us to assess both the impact of current and previous invalid cuing on task repetition performance as well as to compare task switch performance when the task was invalidly cued as a (different) task switch vs. when it was cued as a task repetition. Noteworthy in this regard, in previous studies of cuing validity, in which only two tasks were used (or in which cues were used which could only be followed by two tasks, one of them constituting a task repetition), invalid cuing of a task switch implied an actual task repetition whereas invalid cuing of a task repetition implied an actual task switch. Inasmuch as preparation for task repetitions and switches involves different processes, comparing invalidity costs on task repetition and switch trials is confounded by this factor.

### Method

#### Participants

Five female and 25 male students of the Helmut-Schmidt-University/University of the Federal Armed Forces Hamburg participated in exchange for partial course requirements. They ranged in age from 20 to 27 years.

#### Apparatus and stimuli

Apparatus and stimuli were the same as in Experiment 1 with the exception that responses were given by pressing one of two response keys which were mounted on an external rectangular keyboard (10 cm × 18 cm). The response keys extended 1.0 cm × 1.0 cm and were separated by 8.0 cm (parallel to the keyboards long axis). Participants pressed the response keys with the index or middle fingers of their left and right hand.

### Procedure

The procedure was identical to the procedure of Experiment 1 with the following exceptions. First, the task was chosen randomly on each trial, resulting in an expected proportion of one third task repetition trials. After an incorrect response, the identical trial was repeated. Such repetitions were discarded from the analyses and not counted as trials.

### Results

The same exclusion criteria as in Experiment 1 were applied. Additionally, to ensure identical preparation conditions, we excluded data from trials which were invalidly cued as a task repetition. We also excluded direct stimulus repetitions because these have been shown to facilitate responding selectively on trials in which the task or cue repeats (Hübner et al., 2004b).

**Figure 3:**
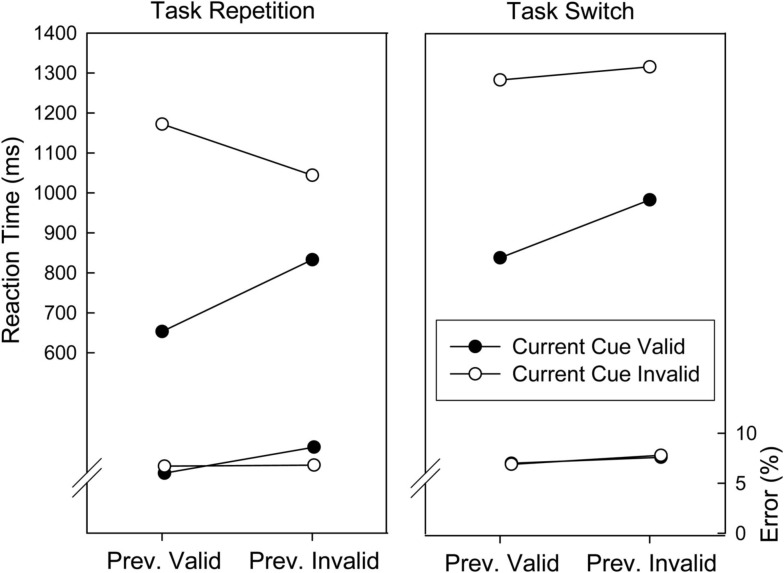
**Mean reaction times and error proportions of Experiment 2 as a function of cuing validity of the current and preceding trials and task sequence**.

In an ANOVA with repeated measures on the factors Validity on Current Trial (valid, invalid), Validity on Preceding Trial (valid, invalid), and Task Sequence (repetition, switch) on the mean RTs, all main effects were significant. There was an invalidity cost of 365 ms, *F*(1, 29) = 222.12, *p* < 0.001, post-invalid slowing of 70 ms, *F*(1, 29) = 21.09, *p* < 0.001, and a task switch cost of 168 ms, *F*(1, 29) = 29.33, *p* < 0.001. The invalidity cost was larger after a valid trial than after an invalid trial (482 vs. 247 ms), *F*(1, 29) = 159.59, *p* < 0.001. Furthermore, the invalidity cost was larger for task switches than for task repetitions (389 vs. 340 ms), *F*(1, 29) = 11.11, *p* < 0.003, and this was further modulated by a three-way interaction involving all factors, *F*(1, 29) = 15.48, *p* < 0.001. As can be seen in Figure 3, the reduction of the invalidity cost after an invalid predecessor trial was more pronounced on task repetition than on task switch trials. To examine this result pattern in more detail, planned comparisons were run, contrasting the invalidity cost on task repetition and task switch trials, separately. Both comparisons reached significance, *F*(1, 29) = 6.37, *p* < 0.02, and *F*(1, 29) = 19.54, *p* < 0.001, respectively, demonstrating that after a validly cued trial the invalidity cost affected task repetitions more strongly than task switches, whereas the opposite pattern occurred after an invalidly cued trial. No significant results were found in the corresponding error analysis (all *p*s > 0.24).

Analogously to Experiment 1, we ran an ANOVA, confined to data from task switch trials following an invalidly cued trial, with the factors Validity on Current Trial (valid, invalid) and Task (Invalidly) Cued on Preceding Trial (different, same as current task). The mean RT and error data are depicted in Figure 2, right panel. Regarding RTs, switching to a task that was invalidly cued on the preceding trial again impaired performance by 97 ms, *F*(1, 19) = 14.60, *p* < 0.001. This impairment did not differ for validly and invalidly cued trials, *F*(1, 19) = 2.33, *p* = 0.138. There were no significant effects in the error analysis (all *p*s > 0.20)^1^.

### Discussion

Experiment 2 replicated the sequential modulation of cuing validity found in Experiment 1 and extended it to task repetition trials. Again, the invalidity cost was confined to RTs, whereas errors were kept at a constant level. Furthermore, Experiment 2 replicated the extra cost when switching to a previously invalidly cued task.

Intriguingly, the reduction of the invalidity cost after invalid trials was further modulated by task sequence. More precisely, task repetitions were associated with a larger invalidity cost after a valid trial and a smaller invalidity cost after an invalid trial than task switches. This modulation cannot be attributed to a difference in the preparation processes because at the time of preparation participants did not know whether a task repetition or switch would follow. By consequence, it has to be assumed that task repetitions are more strongly affected by the variation in cue-based preparation after valid and invalid trials than task switches. At first sight, it seems plausible to assume that a recently instantiated task-set may be more easily re-activated after being misled, thus predicting a lower invalidity cost for task repetitions than for task switches after an invalidly cued trial. However, findings of relative performance impairment when switching back to a task compared to when switching to a task not executed on previous trials (i.e., ABA vs. CBA task sequences) have been accounted for in terms of inhibition of the to-be-abandoned task-set (Mayr and Keele, 2000; Mayr, 2007; for a review see Koch et al., 2010). Assuming that backward inhibition is implemented during preparation, invalidity costs should be larger on task repetition trials (i.e., because inhibition has to be overcome) than on task switch trials. Given the broad empirical evidence that backward inhibition depends on appropriate preparation for the new task (Dreisbach et al., 2002, Experiment 5; Mayr and Keele, 2000; Hübner et al., 2003; Dreisbach and Haider, 2006; Kuhns et al., 2007, Experiment 3; see also Li and Dupuis, 2008), it seems conceivable that backward inhibition suffers from post-invalid reduction of preparatory activity. That is, the reduction of preparation after an invalidly cued trial (which normally goes along with the inhibition of the just executed task) then reduces backward inhibition accordingly. Backward inhibition might thus account for both the larger invalidity cost on task repetition trials after a valid predecessor trial (more invalid preparation and thus stronger backward inhibition on invalid trials), and the smaller invalidity cost on task repetitions after an invalid predecessor trial (less preparation and thus less backward inhibition on invalid trials).

## General Discussion

When people switch between simple cognitive tasks, performance benefits from advance cuing of the identity of the upcoming task. In particular, task performance increases with the probability of occurrence, suggesting that preparation is gradually adjusted to its expected utility. The current study provides evidence for adjustment of task preparation on the basis of its utility on the directly preceding trial. On both task switch (Experiment 1 and 2) and task repetition trials (Experiment 2) costs of invalid task cuing were strongly reduced when the directly preceding trial also involved an invalid cue. This sequential modulation resembles trial-to-trial adjustment effects regarding the processing of task-irrelevant stimulus features (Gratton et al., 1992; Botvinick et al., 2001) and suggests that participants engage less in cue-based task preparation processes after being misled.

Extending previous studies of invalid task cuing, we used a task switching paradigm with three tasks which allowed us to deconfound invalid task cuing and task sequence by assessing performance on both task repetition trials and on task switch trials after cuing of a task switch. Contrasting with previous results of additive or under additive interactions of cuing validity and task sequence (Ruthruff et al., 2001; Dreisbach et al., 2002; Hübner et al., 2004a) we observed an overall larger invalidity cost on task switches than on task repetitions. This interaction is difficult to interpret, however, given the modulation by previous cuing validity, that is, the fact that the reduction of the invalidity cost was more pronounced on task repetition than on task switch trials. A possible explanation is to assume that performance on invalidly cued task repetition trials is particularly impaired by anticipatory backward inhibition and that preparation following an invalidly cued trial lacks this component. Future research is necessary to disentangle preparatory activation of the set for an upcoming task and inhibition of the set of the preceding task in more detail.

In addition to assessing the sequential modulation of the invalidity cost, our experimental set-up allowed us to look more specifically at the performance on task switch trials following an invalidly cued trial when switching to a previously cued task vs. when switching to the remaining task. In both experiments, we observed an extra cost in the former case. This finding can be explained by particular reluctance to prepare for a task (or to use a task cue) which is associated with recent invalid preparation. Alternatively, it is conceivable that invalid task preparation enhances task competition which then triggers control measures of task-set inhibition (Hübner et al., 2004a).The results of the current study thus provide a new example for online, trial-to-trial adjustment of cognitive processing, clearly demonstrating that the degree of task preparation depends on previous success, possibly reflecting both anticipatory and reactive inhibition to ensure efficient performance regarding a currently relevant task in the face of exogenously and endogenously evoked competition.

## Conflict of Interest Statement

The authors declare that the research was conducted in the absence of any commercial or financial relationships that could be construed as a potential conflict of interest.

## Footnotes

^1^To compare consequences of invalid preparation for a task repetition and for a task switch, we conducted an ANOVA involving only invalidly cued task switches with the factors Validity on Preceding Trial and Cuing (Task Repetition, Task Switch). Regarding RTs, trials which were invalidly cued as a task repetition were generally facilitated (by 74 ms after a valid predecessor trial and by 48 ms after an invalid predecessor trial), *F*(1, 29) = 19.54, *p *< 0.001. The two factors did not interact, *F *< 1. There were no significant effects in the corresponding error analysis (all *F*s < 1).
